# A cytokine screen using CRISPR-*Cas9* knock-in reporter pig iPS cells reveals that Activin A regulates *NANOG*

**DOI:** 10.1186/s13287-020-1588-z

**Published:** 2020-02-18

**Authors:** Junjun Xu, Zheng Zheng, Xuguang Du, Bingbo Shi, Jichang Wang, Dengfeng Gao, Qianqian Zhu, Xinze Chen, Jianyong Han

**Affiliations:** 1 State Key Laboratory for Agrobiotechnology, College of Biological Sciences, China Agricultural University, Beijing, 100193 China; 2 College of Biological Sciences, China Agricultural University, Beijing, 100193 China; 3 Center for Stem Cell Biology and Tissue Engineering, Key Laboratory for Stem Cells and Tissue Engineering, Ministry of Education, Sun Yat-senUniversity, Guangzhou, Guangdong 510080 China; 4 Department of Histology and Embryology, Zhongshan School of Medicine, Sun Yat-sen University, Guangzhou, Guangdong 510080 China

**Keywords:** Pluripotency, CRISPR/*Cas9*, *NANOG* reporter, Cytokine screen, Activin A

## Abstract

**Background:**

*NANOG* functions as the gateway for the generation of pluripotent stem cells (PSCs) in mice and humans. NANOG is a transcription factor highly expressed in pig pre-implantation embryos, indicating that it is a conserved pluripotency-associated factor. However, pig *NANOG* reporter PSCs have yet to be established, and the regulation of pluripotency by *NANOG* is not fully understood in this animal.

**Methods:**

In this study, pig *NANOG* tdTomato knock-in reporter positive PC-iPS cells were established using CRISPR*/Cas9*. The resulting cell line was treated with several cytokines and their corresponding inhibitors to identify pathways that regulate *NANOG* expression. The pathways examined were LIF (leukemia inhibitory factor)/IL6 (interleukin 6)-STAT3, FGF (fibroblast growth factor)/ERK, IGF1 (insulin-like growth factor 1)/PIP3 (phosphoinositide 3-kinase)-AKT, Activin A/SMAD, and BMP4 (bone morphogenetic proteins)/SMAD.

**Results:**

Our experiments showed that the Activin A/SMAD pathway is directly associated with activation of *NANOG* expression in the pig, as is also the case in mice and humans. Activin A directly regulates the expression of pig *NANOG* via SMAD2/3; inhibition of this pathway by SB431542 resulted in inhibition of NANOG expression.

**Conclusions:**

Our results show that Activin A plays an important regulatory role in NANOG-mediated pluripotency in pig iPS cells. Activin A treatment may be therefore an effective method for de novo derivation of authentic embryonic stem cells (ESCs) from pig pre-implantation embryos.

**Electronic supplementary material:**

The online version of this article (10.1186/s13287-020-1588-z) contains supplementary material, which is available to authorized users.

## Background

The availability of mouse [1] and human [2] embryonic stem cells (ESCs) has stimulated advances in regenerative medicine and provided insights into the genes that control pluripotency and cell fate. *NANOG*, *OCT4*, and *SOX2* are key regulatory genes that encode the core pluripotency circuitry in mice, rats, and humans [3, 4]. NANOG is a transcription factor that plays an important role in maintaining the pluripotency of ESCs [5, 6]; it safeguards pluripotency and mediates germline development in mice [7]. Downregulation of *NANOG* can induce human ESC differentiation [8]. NANOG is also expressed heterogeneously: high NANOG expression is observed in ESCs, whereas low expression is observed in primitive endoderm cells [9]. NANOG is also highly expressed in pig pre-implantation embryos [10]. Recently, pig pluripotent stem cells (PSCs) were established from the inner cell mass of pig blastulas [11–13]. We found that induced pluripotent stem cells (iPSCs) from pigs express NANOG heterogeneously [14], as in mouse PSCs [15, 16]. Various CRISPR*/Cas9* gene-editing strategies have been used to create reporter cell lines that accurately represent NANOG expression dynamics [16–20]. However, pig *NANOG* knock-in reporter PSCs have not been established.

ESC fate determination is controlled by several key signaling pathways; LIF/JAK-STAT, FGF/ERK, Activin/SMAD, BMP4/SMAD, and IGF1-PI3K/AKT. Mouse and human ESCs use different regulatory pathways for sustaining pluripotency. Mouse pluripotency is defined as having naïve and primed states [21]. Mouse ESCs (naïve state) rely on the LIF/JAK-STAT pathway to maintain pluripotency. However, epiblast stem cells (EpiSCs) from post-implantation blastocysts (primed state) rely on the FGF/ERK and Activin/SMAD signaling pathways [22]. IL6 also activates the JAK-STAT3 pathway and increases the induction efficiency for mouse and human pluripotent stem cells (iPS) [23]. In contrast, human ESCs rely on the bFGF and TGF-β/Activin signaling pathways to maintain their pluripotency [24]. The BMP4-SMAD and LIF-STAT pathways sustain mouse ESC pluripotency [25], while the BMP4-SMAD pathway promotes human ESC differentiation by downregulating *NANOG* [24, 26, 27]. The IGF1-PI3K/AKT signaling pathway promotes human pluripotency and self-renewal [28, 29] and also promotes mouse pluripotency [30, 31]. Whether pig *NANOG* regulation is similar to that of mouse or human is unknown.

Pig pluripotent stem cells can be used in pig breeding, to model pig disease and to test pre-clinical regenerative medicines. Although pig expanded pluripotent stem cells [12], dome-shaped iPS cells [14], and ESCs [13] have recently been established, germline ESCs/iPSCs are not yet available. Many cytokines have been used to generate pig pluripotent stem cells, such as LIF [32–34], bFGF [35], and LIF and bFGF in combination [36, 37]. However, it is not known which cytokine directly activates *endo-NANOG* expression*.*

To address this question, we established pig *NANOG* tdTomato knock-in iPS cells using CRISPR*/Cas9* and then treated them with various cytokines and their corresponding inhibitors to identify the key pathway that regulates *NANOG* tdTomato expression.

## Materials and methods

### Nucleic acid extraction and PCR/RT-PCR

Total DNA and RNA were extracted from cultured pig *NANOG* tdTomato knock-in positive PC-iPS cells using DNA and RNA extraction kits following the manufacturer’s protocol (Tiangen, DP304-03 and DP430, respectively). cDNA was prepared by reverse transcription PCR using 5× All-In-One RT MasterMix (Abcam, G916). Quantitative PCR was conducted in 15 μL reactions containing 2× RealStar Green Power Mixture (Genestar, A311-05), using a quantitative PCR instrument (Roche, LightCycler 480). The amplification conditions were as follows: 95 °C for 10 min; then 45 cycles of 95 °C for 10 s, 60 °C for 10 s, and 72 °C for 10 s.

### Vector construction

A custom single-guide RNA (sgRNA) sequence was designed using the Benchling web tool (https://benchling.com/crispr). The *NANOG* sgRNA sequence was inserted into the pGL3-U6-sgRNA-PGK-puromycin plasmid (Addgene, 51133) using the NEB® Golden Gate Assembly Kit (BsaI-HF®v2) according to the manufacturer’s instructions (NEB, E1601). The structure of the *NANOG* sgRNA plasmid is shown in Additional file 2: Figure S1A. The sgRNA sequence was inserted in the form of an annealed oligonucleotide primer (Additional file 1: Table S1: NANOG sgRNA F; NANOG sgRNA R) containing sticky ends. The vector structure was verified by Sanger sequencing.

The DNA donor plasmid was constructed using NEBuilder® HiFi DNA Assembly Master Mix (NEB, E2621X) and consists of four fragments (Backbone, Left Homology Arm-2×FLAG, 2×FLAG-P2A-tdTomato-loxp-Puro-loxp, Right Homology Arm). The structure of the *NANOG* donor plasmid is shown in Additional file 2: Figure S1B. The backbone fragment was generated by restriction enzyme digestion (XbaI and HindIII) and the 15–25-bp identical sequence required for Gibson assembly was generated using 5′-overhang primers. The homology arm fragments were PCR amplified in three steps. First, from chromosome 1 (Chr1), a 5196-bp region containing *NANOG* coding sequences and downstream region (Additional file 1: Table S1: *NANOG* Raw F; *NANOG* Raw R) was subcloned. Second, the left and right homology arms were amplified from the 5196-bp template using an overhang primer with a 23-bp gRNA target upstream of the left arm (Additional file 1: Table S1: *NANOG* 5′ Arm 1F; *NANOG* 5′ Arm 1 *Mut* R) and downstream of the right arm (Additional file 1: Table S1: *NANOG* 3′ Arm 1F; *NANOG* 3′ Arm 1R). The left arm’s silent point mutation (*Mut*) was within the gRNA target protospacer adjacent motif (PAM) in order to prevent *Cas9* from cutting after insertion or introducing an unintended cut on the donor strand. Finally, the 15–25-bp identical sequences were added to overhang primers using amplicons from the second step as templates. Specifically, for the region downstream from the left arm, the identical downstream utilized sequence was the 24-bp FLAG end introduced by an overhang primer containing 2×FLAG in two steps (Additional file 1: Table S1: *NANOG* 5′ Arm 2 F; *NANOG* 5′ Arm 2 R; *NANOG* 5′ Arm 3 F; *NANOG* 5′ Arm 3 R). For the right arm, the identical end was added in one step. The tdTomato sequence and puromycin resistance cassette were PCR amplified from an existing plasmid as a 3-kb P2A-tdTomato-loxp-Puro-loxp fragment, which was then used as a template (Additional file 1: Table S1: tdTomato-Puro 1F; tdTomato-Puro 1R). Overhang primers containing 2×FLAG sequence and downstream identical sequence were added upstream and downstream of the 3-kb fragment (Additional file 1: Table S1: tdTomato-Puro 2F; tdTomato-Puro 2R). The four fragments with specific identical ends were mixed at a molar ratio of backbone: left arm: tdTomato and puromycin: right arm = 1:2:2:2, and incubated at 55 °C for 1 h, following the protocol in the NEB HiFi-DNA assembly instruction manual. The product was transformed into Trans5-α competent cells (Transgene, CD201-02). The construct was verified by Sanger sequencing.

### Genotyping

Genomic DNA was extracted from cells containing the tdTomato *NANOG* insertion. To verify the target insertions, PCR was used to amplify the 5′ and 3′ junctions from the Chr1-*NANOG* and Chr5-*NANOG* transgenes; WT PC-iPS genomic DNA was used as control. The genotyping primers were intentionally designed to anneal outside of the homology arm as a precaution against the entire linearized donor being accidentally inserted by non-homologous end joining (NHEJ) during DNA repair. However, no evidence for this event was detected in later experiments (Additional file 1: Table S1: Chr1 5′ Test F; Chr1 5′ Test R; Chr5 3′ Test F; Chr5 3′ Test R; Chr1 3′ Test R; Chr1 5′ Test F; Chr1 5′ Test R). Insertions were observed at both targeted sites. The PCR-amplified DNAs were subjected to Sanger sequencing to confirm the integrity of the construct.

### Cell culture and plasmid electro transfection

Pig pericyte-derived induced pluripotent stem cells (PC-iPS cells) were cultured in a modified EPS culture system [14]. Cells were cultured in LCDMV, which consisted of a base medium containing 50% (v/v) DF12 (Gibco;10,565–018) and 50% (v/v) Neurobasal™ Medium (Gibco, 21103-049). The medium also contained 10 ng/ml LIF (Peprotech, 300-05-1000), 1 μM CHIR99021 (CHIR) (Tocris, 4423), 2 μM (S)-(+)-dimethindene maleate (DIM) (Tocris, 1425), 2 μM minocycline hydrochloride (MIH) (Santa Cruz, sc-203,339), and 40 μg/mL vitamin C (Vc) (Sigma, A92902). When the PC-iPS cells reached 70% confluence, they were dissociated using StemPro™ Accutase™ Cell Dissociation Reagent (A1110501; Gibco). Electrotransfection was used to transfer plasmids into the cells. Briefly, 4 μg pST1374-NLS-flag-linker-Cas9 plasmids (Addgene, 44,758), 4 μg *NANOG* sgRNA plasmid, and 4 μg *NANOG* HMEJ donor plasmids (mass ratio 1:1:1) were co-transfected into 2.5 × 10^6^ PC-iPS cells using a Lonza AMAXA Lonza Nucleofector 2B/II (Loza, Amaxa 2B), configured to use the A030 program. To select transformants, puromycin dihydrochloride (Thermor, A1113803) (1 μg /mL) and blasticidin (Invitrogen, R210–01) (10 μg /mL) were added to the culture media 24 h post transfection. After 48 additional hours of incubation, puromycin dihydrochloride (1 μg/mL) was added again, the cells were cultured for four additional days.

### Immunocytochemistry

PC-iPS cells were passaged in 24-well plates (Nunc, 142475) and cultured for 3 days in preparation for immunocytochemistry analysis. Cells were fixed by aliquoting 4% paraformaldehyde solution (Sangon Biotech, 3053589-4) into each well then incubating 20 min at room temperature. Cells were then washed twice with PBS (Gibco, C14190500BT), then treated with 0.5% Triton X100 (Sangon Biotech, A110694-0100) in PBS for 40 min at room temperature. Cells were washed twice with PBS then blocked with a blocking reagent (Beyotime, P0102) for 30 min at room temperature. Next, primary antibodies against NANOG protein (Peprotech, 500-P236) (1:500), SOX2 protein (Santa Cruz, SC365823) (1:500), OCT4 protein (Santa Cruz, SC8628) (1:500), α-SMA protein (Abcam, ab5694) (1:500), Vimentin protein (Abcam, ab92547) (1:500), and β-Tubulin protein (Abcam, ab45939) (1:500) and ANTI-FLAG® M2 protein (Sigma, F1804-1) (1:1000) were added to appropriate wells and incubated overnight at 4 °C. Cells were washed again, secondary antibodies were added, and the reactions were incubated for 1 h at room temperature. The secondary antibodies were Alexa Fluor 594 donkey anti-mouse IgG (H+L) highly cross-adsorbed secondary antibody (1:750) (Thermo, A21203), Alexa Fluor 594 donkey anti-rabbit IgG (H + L) highly cross-adsorbed secondary antibody (1:750) (Thermo, A21207), and Alexa Fluor 488 donkey anti-Rabbit IgG (H+L) highly cross-adsorbed secondary antibody (1:750) (Thermo, A-21206). The antibodies were diluted using secondary antibody solution (Beyotime, P0108). DAPI (4′,6-diamidino-2-phenylindole, dihydrochloride) (1:5000) (Invitrogen, D1306) was used to stain nuclei.

### RNA sequencing and transcriptome analysis

Before RNA extraction, feeder cells were removed in order to avoid sequencing artifacts. Dissociated PC-iPS cells were plated onto six-well plates and cultured for 1 h. The suspended cells were collected for RNA extraction, leaving behind the attached cells (primarily consisting of feeders). Total RNA samples (2 μg each) were suspended in 15 μL RNAase-free ddH_2_O, packed in dry ice, and submitted to Anoroad Gene Technology Corporation (Beijing, China) for RNA sequencing.

Low-quality reads and adaptor sequences were discarded using Trim Galore (https://www.bioinformatics.babraham.ac.uk/projects/trim galore/). The remaining reads were aligned to the pig genome version Ssc11.1 (from Ensemble) using Hisat2 [38]. Read counts were calculated using featureCounts [39] and expression levels were normalized as FPKM using gene annotation files from Ensemble as a guide (release 97) and StringTie [40]. Differentially expressed genes (DGE) were identified using DEseq2 [41]. A gene was defined as differentially expressed if its fold-change value was 1 or more, with an adjusted *p* value ≤ 0.05. KEGG pathway analyses were performed using ClusterProfiler [42]. Re-analyzed previously published data are available under the accession codes GSE139512 [43] for pig pre-implantation embryos and E-MTAB-7253 [12] for pig EPS cells.

### Effect of cytokines and their inhibitors on regulation of N*ANOG* tdTomato expression

Media used in these experiments, designated “T1”, was LCDMV (used for culturing PC-iPS) minus LIF. Experiments were conducted in 12-well plates (Nunc; 150628), and *NANOG* tdTomato PC-iPS cells were passaged three times to ensure that *NANOG* tdTomato was stably expressed. To test the response of each cell line to individual cytokines, the T1 culture medium was supplemented with LIF (Peprotech, 300–05-1) (5 ng/mL, 10 ng/mL), IL-6 (R&D, 206-IL) (50 ng/mL, 100 ng/mL), IGF1 (Peprotech, 100-11-1000) (50 ng/mL, 100 ng/mL), bFGF (Peprotech, D2017) (5 ng/mL, 10 ng/mL), Activin A (Peprotech, 1017) (5 ng/mL, 10 ng/mL), or BMP4 (Peprotech, 120-05ET) (5 ng/mL, 10 ng/mL). The corresponding signal pathway inhibitors were as follows: 10 μM ruxolitinib (INCB018424) (Selleck, S1378) (Jak-STAT pathway inhibitor), 10 μM Ly294002 (S1737 1 mg) (PIP3-AKT pathway inhibitor), 10 μM AZD4547 (Selleck, S2801) (bFGF-ERK pathway inhibitor), 10 μM SB431542 (Selleck, S1067) (TGF-β/Activin A pathway inhibitor), and Noggin (R&D, 6057-NG-025) (antagonist of BMP4) (50 ng/mL and 100 ng/mL). Fluorescence microscopy, flow cytometry, and RT-PCR were done to detect expression of *NANOG* tdTomato.

### Flow cytometry

*NANOG* tdTomato PC-iPS monolayers were dissociated into single cells using StemPro™ Accutase™ Cell Dissociation Reagent. To prevent impurities from affecting the fluorescence signal, the cells were filtered through strainer meshes (Corning, 431751) and washed twice with PBS. Fluorescence-activated cell sorting was performed using a BD FACSAria at the Tsinghua Core Facility Center. The BD FlowJo_V10 application was used to analyze the data.

### Western blots

Cells were washed two times with cold PBS, and then dissociated and centrifuged at 3000 rpm for 5 min at 4 °C. Two hundred microliters of RIPA Lysis Buffer (Beyotime Biotechnology, P0013K), containing protease and phosphatase inhibitor cocktail (Beyotime Biotechnology, P1045) was added to cell pellets which were then incubated on ice as they lysed. Cell lysates were centrifuged at 14,000×*g* for 25 min at 4 °C, and the protein concentration in the supernatants was determined by Bradford assay (BIO-RAD, 500-0205). Separating gel buffers for 5% and 10% SDS-PAGE were prepared (CWBIO, CW0026), and samples were subjected to electrophoresis at 80 V for 5 min and 120 V for 45 min. Proteins were transferred to PVDF membrane (IPVH00010, Immobilon) at 350 mA for 90 min. Primary antibodies against GAPDH (CST, 2118 L), pSMAD2/3 (CST, 8828 s), SMAD2/3 (CST, 8685p), and Anti-Flag® (Sigma, F1804) were diluted to 5% (mg/v) in a solution of NON-Fat Powdered Milk (Sangon Biotech, A600669-0250). Membranes were incubated with the primary antibodies overnight at 4 °C. Unbound primary antibody was removed by washing membranes three times with TBST. Goat anti-rabbit IgG-HRP (Absin, abs20002A) and goat anti-mouse IgG-HRP (Absin, abs20001) diluted to 5% (mg/v) in a solution of NON-fat powdered milk were used as secondary antibodies. The membranes were incubated with the secondary antibodies for 60 min at room temperature.

## Results

### Generation of pig *NANOG* tdTomato knock-in reporter piPS cells

**Fig. 1:**
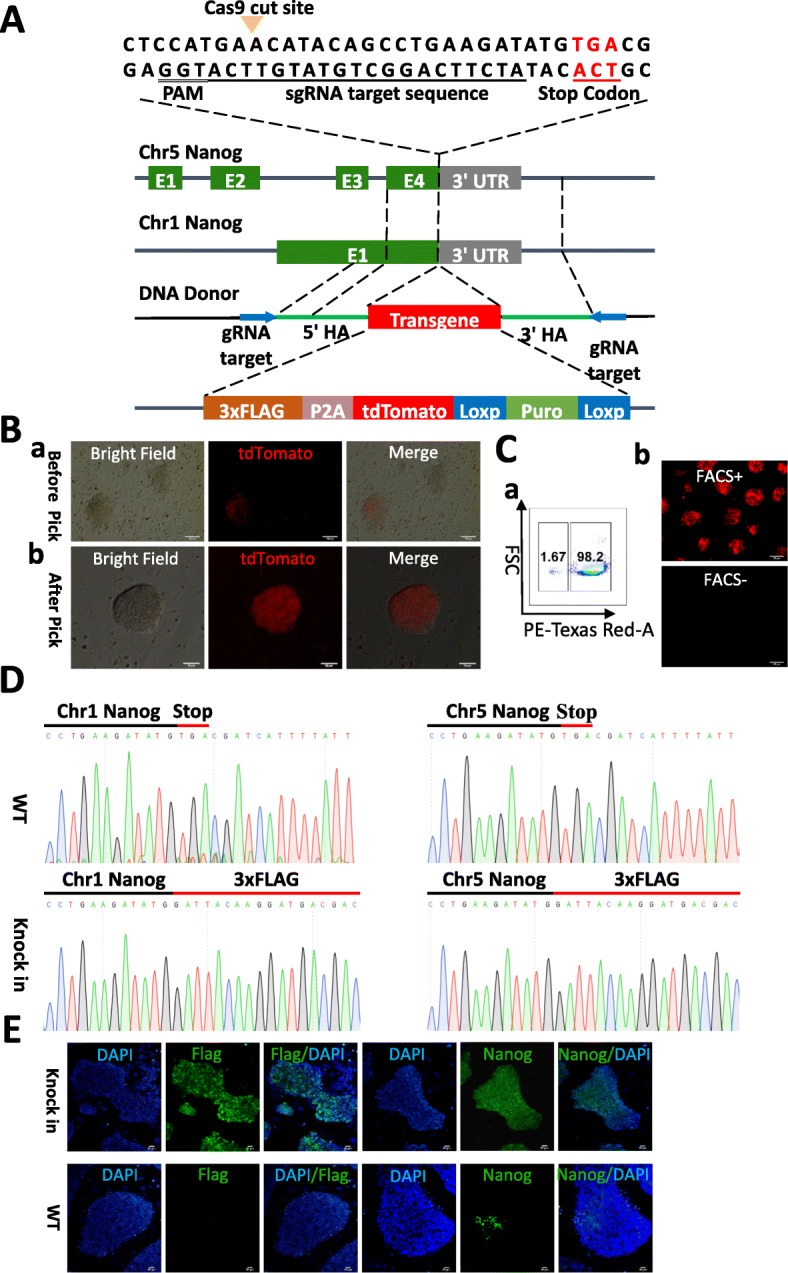
Generation of *NANOG* tdTomato knock-in reporter positive piPS cells. **A** Overview of the strategy for constructing *NANOG* tdTomato knock-in positive cells. The target sequences of *NANOG* on Chr1 and Chr5 are identical. **B** Analysis of *NANOG* tdTomato knock-in positive colonies. (a) Before colonies were picked. (b) After colonies were picked and regrown. Scale bar 50 μm. **C** FACS analysis of *NANOG* tdTomato knock-in positive cells. (a) FACS tdTomato positive cells. (b) Fluorescence micrograph of cells sorted by FACS. Scale bar 200 μm. **D** Sanger sequencing results for the target locus and insert junctions for chromosomes 1 and 5. **E** Immunocytological analysis of NANOG tdTomato knock-in positive cells. *NANOG* tdTomato knock-in positive cells were positive for FLAG and NANOG protein; WT PC-iPS cells were negative for FLAG and heterogeneous for NANOG. Scale bar 25 μm

Because identical target sequences occur within the *NANOG* loci on pig chromosomes 1 and 5, the knock-in strategy is theoretically able to target both loci for integration using only one vector. The target sequence, as well as the DNA donor vector are shown in Fig. 1A. The DNA donor contained a transgene cassette with left and right homology arms (5′ and 3′ HA), flanked by the same gRNA target and PAM sequences.

After integration, the resulting transgene is expected to have the structure 3×FLAG-P2A-tdTomato-Puro. The puromycin resistance gene is driven by its own hPGK promoter and is flanked by two LoxP sequences.

The scheme for generating the *NANOG* tdTomato knock-in reporter PC-iPS is shown in Additional file 3: Figure S2A. Briefly, transfected cells were selected by incubating in medium containing puromycin dihydrochloride (1μg/mL) and blasticidin (10 μg/mL) for 2 days. On the third day of culture, the media was replaced and only puromycin dihydrochloride was added. Every 2 days, the culture medium was replaced, until the seventh day, when the selection medium was replaced with LCDMV. On the 11th day of culture, the colonies exhibited tdTomato expression, while control cells were negative (Fig. 1B, a). Ten independently transfected cultures exhibited tdTomato expression, and microscopic examination showed that individual cells from each culture were able to generate tdTomato dome-shaped colonies (Fig. 1B, b). From these ten, we chose three of the cell lines, designated nanog1, nanog2, and nanog3, for further study.

Because the cultures were generated using heterogeneous populations of transformed cells, individual cells within each culture may be heterozygous or homozygous for the insertion at each allele, contain different combinations of insertions at the two loci, or be completely wild-type. tdTomato gene expression in these cell lines was validated using FACS, PCR, Sanger sequencing, and immunocytological assays. Cells were cultured for three passages and then analyzed using FACS to sort tdTomato-positive cells (Fig. 1C, a). The transfected cells were positive for tdTomato, while the negative control exhibited no signal (Fig. 1C, b). To verify the insertions, the 5′ and 3′ junctions corresponding to the expected insertions at both loci were analyzed by PCR analysis of genomic DNA from candidate knock-in cell lines (Additional file 3: Figure S2B). The genotyping primers were designed to anneal at positions external to the HAs in case the entire linearized donor had been inserted by NHEJ. Sequencing of the amplified junction regions using template DNA from each positive culture revealed that the insertions were seamless and that no mutations were present within the 3×FLAG tag (Fig. 1D). *NANOG* tdTomato knock-in positive cells expressed high levels of NANOG and FLAG, while wild-type PC-iPS expressed heterogeneous NANOG and no detectable FLAG (Fig. 1E). Together, these results demonstrate that we have successfully generated tdTomato *NANOG* knock-in reporter cells.

### Verification and transcriptome analysis of NANOG tdTomato knock-in positive PC-iPS cells

**Fig. 2:**
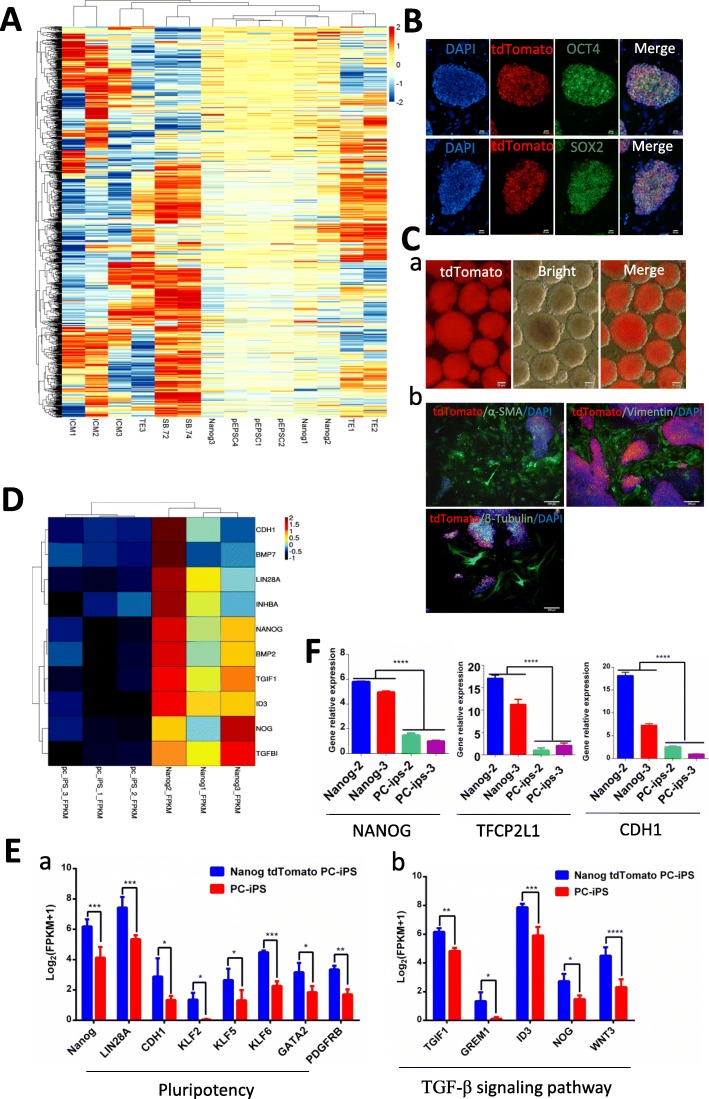
Verification and transcriptome analysis of *NANOG* tdTomato knock-in and WT PC-iPS cells. **A** NANOG tdTomato knock-in positive PC-iPS cells (nanog) could be clustered with pig EPS cells (pEPSC), but separate with trophoblast cells (TE), inner cell mass (ICM), and early blastocysts (SB). **B** Knock-in positive PC-iPS cells were expressed OCT4 and SOX2 pluripotent markers. Scale bar, 20 μm. **C** (a) knock-in positive PC-iPS cells formed EB spheres. Scale bar, 50 μm; (b) knock-in positive PC-iPS cells were differentiated to three germ layers which were ectodermal (β-TUBULIN), mesodermal (α-SMA), and endodermal (VIMENTIN). Scale bar, 200 μm. **D** Heat map of the clustering analysis for expression of selected *TGF-β* signal pathway-related genes in *NANOG* tdTomato knock-in versus WT PC-iPS cells. **E** (a) Pluripotency-related genes were up-regulated in *NANOG* tdTomato knock-in positive PC-iPS cells comparing with WT PC-iPS; (b) *TGF-β* signal pathway-related genes were also upregulated in *NANOG* tdTomato knock-in positive PC-iPS cells. Values were normalized as log_2_(FPKM+1), where FPKM is fragments per kilobase of exon per million reads mapped. **F** RT-PCR analysis of expression of *NANOG*, *TCFP2L1*, and *CDH1* in *NANOG* tdTomato knock-in and WT PC-iPS cells*.* ****< 0.0001; ***< 0.001; **< 0.01, *< 0.5; ns, not significant

The pluripotency of *NANOG* tdTomato knock-in positive PC-iPS cells was corroborated in vitro with the following assays*. NANOG* knock-in positive PC-iPS cells were positive to AP staining (Additional file 3: Figure S2C), the method of AP staining is reference for our publication data [14]. Clustering analysis showed that knock-in positive PC-iPS cells could be clustered with pig EPS cells [12], but were separate from trophoblast cell (TE), inner cell mass (ICM), and early blastocyst (SB) [43] (Fig. 2A) (further detail provided in Additional file 4: Table S2); knock-in positive PC-iPS were expressed OCT4 and SOX2 pluripotent markers (Fig. 2B); knock-in positive PC-iPS also formed EB sphere (Fig. 2C, a) and were differentiated to three germ layers which were ectodermal (β-TUBULIN), mesodermal (α-SMA), and endodermal (VIMENTIN) (Fig. 2C,b).

In order to determine how NANOG expression affects RNA expression, we performed high-throughput RNA sequencing on *NANOG* tdTomato knock-in positive and WT PC-iPS cells and then identified genes that were differentially expressed (log_2_[fold-change ≥1]; adjusted *p* value ≤ 0.05). The results are summarized as a volcano plot in Additional file 5: Figure S3A (further detail is provided in Additional file 6: Table S3). A total of 633 transcripts were more abundant, and 536 were less abundant, in cells containing the *NANOG* tdTomato knock-in compared with WT PC-iPS. A clustering analysis suggests that the expression levels for many genes changed as a result of the *NANOG* tdTomato knock-in positive (Additional file 5: Figure S3B). The differentially expressed genes were also screened using the KEGG pathway database (Additional file 7: Table S4) (adjusted *p* value<0.05). These analyses revealed that the TGF-β, Hippo, PIP3-AKT, and Wnt signal pathways, all of which are related to stem cell pluripotency, are associated with the differentially expressed genes listed in Additional file 8: Figure S4. Cluster analysis for expression of the TGF-β signal pathway-related genes is shown in Fig. 2D. Pluripotency-related genes were upregulated in *NANOG* tdTomato knock-in positive PC-iPS cells, including *NANOG*, *LIN28A*,*CDH1*, *KLF2*, *KLF5*, *KLF6*, *GATA2*, and *PDGFRB* (Fig. 2E, a); TGF-β signal pathway-related genes were also upregulated in *NANOG* tdTomato knock-in positive PC-iPS cells, including *TGIF1*, *GRIM1*, *ID3*, *NOG*, and *WNT3* (Fig. 2E, b). Results from RT-PCR analysis confirmed that expression of *NANOG*, *TFCP2L1*, and *CDH1* is upregulated in *NANOG* tdTomato knock-in positive cells (Fig. 2F). Together, the data demonstrate that *NANOG* tdTomato knock-in positive PC-iPC cells maintain pluripotency and upregulate the TGF-β signal pathway.

### Analysis of media components on *NANOG* expression

**Fig. 3:**
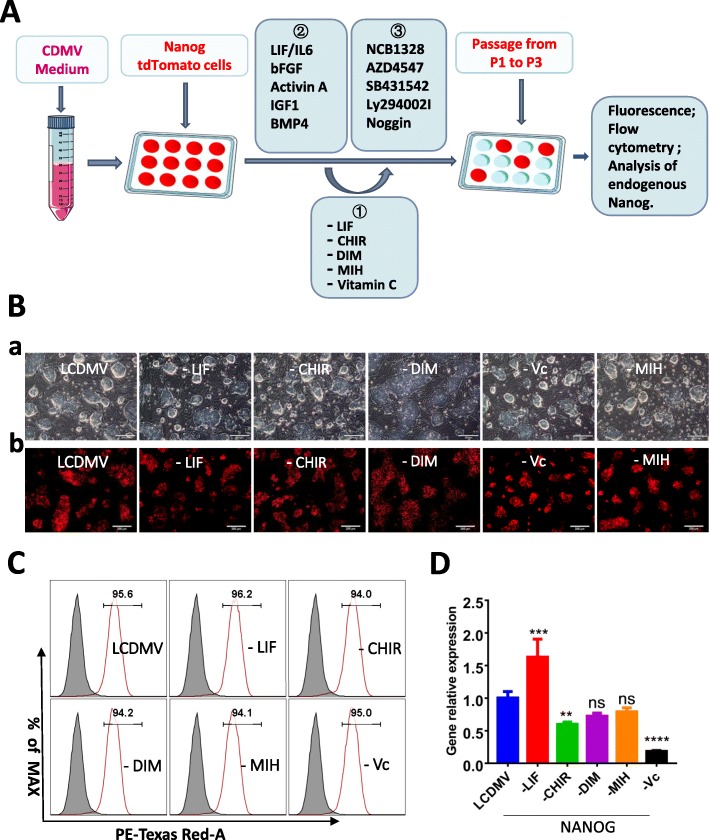
Effects of base medium components on *NANOG* regulation. **A** Experimental design for testing the effects of medium components, cytokines, and inhibitors on *NANOG* tdTomato regulation. **B** Light and fluorescence microscopy images showing expression of tdTomato *NANOG* in cells grown in LCDMV from which individual medium components have been removed. (a) When DIM was removed from the media, light microscopy showed that the cells differentiated to a flatted state, cells in all other media were dome-shaped; (b) fluorescence microscopy revealed that tdTomato *NANOG* was expressed in all media. Scale bar 200 μm. **C** Flow cytometric analysis of tdTomato cells grown in LCDMV from which individual medium components have been removed. **D** RT-PCR analysis of *NANOG* mRNA expression in tdTomato cells grown in LCDMV from which individual medium components have been removed. ****< 0.0001; ***< 0.001; **< 0.01, *< 0.5; ns, not significant

A schematic of the experimental design for testing medium components, cytokines, and inhibitors for their effect on *NANOG* tdTomato regulation is presented in Fig. 3A. In order to examine the effects of inhibitors and activators on the expression of *NANOG* tdTomato, it was first necessary to identify a culture medium that would not introduce artifacts into the experimental results. Components in the LCDMV culture medium (LIF, CHIR, DIM, MIH, and Vc) were removed one at a time. Cells were cultured for three passages in each drop-out medium and tested for expression of tdTomato *NANOG* using fluorescence microscopy, flow cytometry, and RT-PCR. When DIM was removed, light microscopy showed that the cells had a flatted and differentiated morphology (Fig. 3B, a). Fluorescence microscopy revealed that tdTomato *NANOG* was expressed in all media (Fig. 3B, b), and flow cytometry showed that tdTomato cells constituted over 90% of the cells in each culture (Fig. 3C). RT-PCR analysis showed that removal of LIF increased *NANOG* expression, while the removal of CHIR and Vc reduced it. DIM and MIH did not influence *NANOG* expression (Fig. 3D). Therefore, CDMV (designated T1) was used as a base medium in the experiments described below.

### Analysis of *NANOG* activation by cytokines

**Fig. 4:**
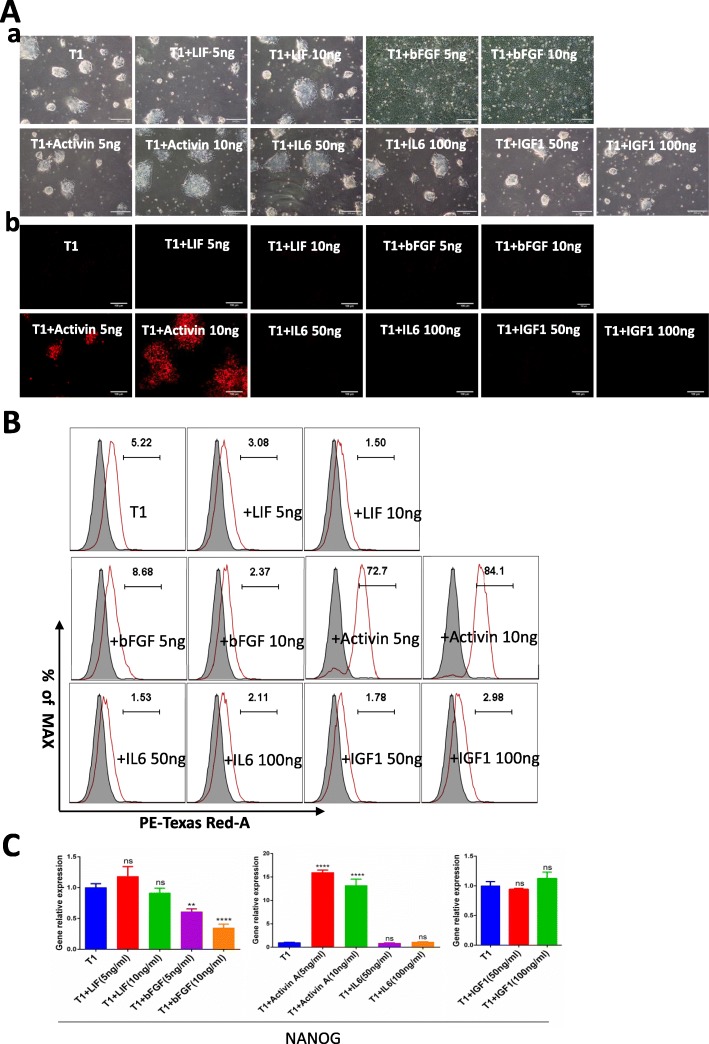
Activation of NANOG expression by various cytokines. **A** Images obtained using optical and fluorescence microscopy showing NANOG expression in *NANOG* tdTomato knock-in positive cells grown in the presence of different cytokines. Growth in T1 medium alone was used as a negative control. (a) Light microscopy showed that cells treated with bFGF had a flattened and differentiated morphology, cells treated wilt other cytokines were dome-shaped. Scale bar 200 μm; (b) examination by fluorescence microscopy showed that only cells exposed to Activin A expressed tdTomato. Scale bar 100 μm. **B** Flow cytometric analysis of *NANOG* tdTomato knock-in positive cells cultured in the presence of different cytokines. **C** RT-PCR analysis of RNA harvested from *NANOG* tdTomato knock-in cells cultured in the presence of different cytokines. ****< 0.0001; ***< 0.001; **< 0.01, *< 0.5; ns, not significant

To determine whether *NANOG* pluripotency is regulated the same way in pigs as in mice and humans, we examined the LIF/IL6-STAT3, FGF-ERK, IGF1/PIP3-AKT, and Activin-SMAD pathways to test the effects of cytokines on *NANOG* tdTomato expression. Because feeder cells secrete many cytokines that can influence *NANOG* expression, we conducted the cytokine screens in a feeder-free culture system using plates coated with Matrigel (BD, 354277). *NANOG* tdTomato knock-in positive were cultured for three continuous passages in the presence of LIF, IL6, IGF1, bFGF, or Activin A in T1 culture medium. After three passages, cells were visualized through optical microscopy, flow cytometry, and RT-PCR analysis. Light microscopy showed that cells treated with bFGF had a flattened and differentiated morphology, while cells treated with other cytokines were dome-shaped (Fig. 4A, a). Examination by fluorescence microscopy showed that only cells exposed to Activin A expressed tdTomato (Fig. 4A, b). This result was confirmed by flow cytometry, which showed that cultures receiving Activin A (at 5 and 10 ng/ml) contained 72.7% and 84.1% tdTomato-positive cells, respectively, while other treatments produced few if any positive cells (Fig. 4B). We also measured expression of *NANOG* mRNA in the third passage cells. RT-PCR analysis showed that Activin A treatment was associated with a significantly higher *NANOG* expression compared to other cytokines; the lowest *NANOG* expression was in bFGF treated cells (Fig. 4C). In conclusion, Activin A activates *NANOG* expression in pig PC-iPS cells.

### Expression of NANOG in response to cytokine pathway inhibitors and BMP4

**Fig. 5:**
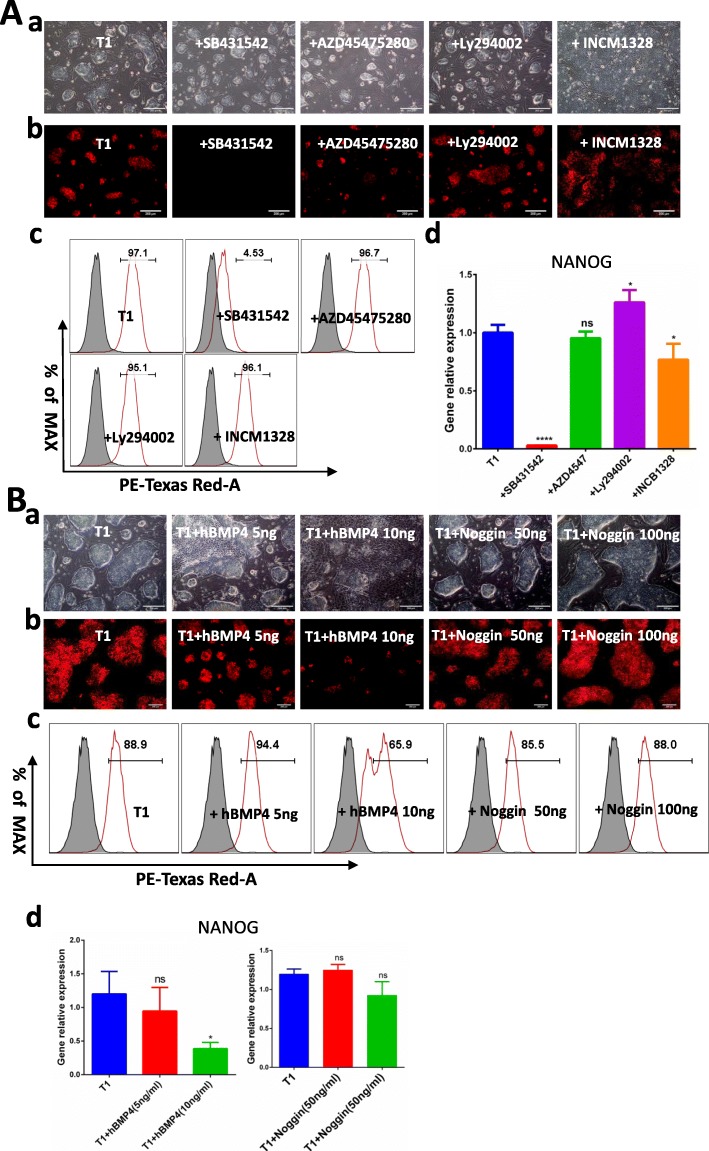
Expression of NANOG in response to cytokine inhibitors and hBMP4. **A** Optical and fluorescence microscopy showing NANOG expression in *NANOG* tdTomato knock-in positive cells grown in the presence of cytokine inhibitors. (a) Light microscopy showed that *NANOG* tdTomato PC-iPS cells were dome-shaped in all the groups. Scale bar 200 μm. (b) Fluorescence microscopy showed that only SB431542 detectably inhibited *NANOG* tdTomato expression. Scale bar 200 μm. (c) Flow cytometric analysis of *NANOG* tdTomato knock-in positive cells cultured in the presence of inhibitors. (d) RT-PCR analysis of RNA harvested from *NANOG* tdTomato knock-in positive cells cultured in the presence of inhibitors. **B** Flow cytometric and RT-PCR showing *NANOG* expression in *NANOG* tdTomato knock-in positive cells grown in the presence of BMP4 and Noggin. (a) Light microscopy showed that most of the cells treated with 10 ng/ml hBMP had differentiated, but cells in the control and antagonist groups had not. Scale bar 200 μm. (b) Fluorescence microscopy also showed that cells treated with 10 ng/ml hBMP had markedly lower expression of *NANOG* tdTomato, compared with cells cultured in T1 alone and cells receiving antagonists. Scale bar 200 μm. (c) Flow cytometric analysis of *NANOG* tdTomato knock-in positive cells cultured in the presence of BMP4 and Noggin. (d) RT-PCR analysis of RNA harvested from *NANOG* tdTomato knock-in positive cells cultured in the presence of BMP4 and Noggin. ****< 0.0001; ***< 0.001; **< 0.01, *< 0.5; ns, not significant

To further examine the pathways that potentially regulate *NANOG* expression, we cultured cells in the presence of INCB1328 (an inhibitor of the LIF/IL-6 STAT-JNK3 pathway), AZD4547 (an inhibitor of the bFGF/ERK pathway), Ly294002 (an inhibitor of the IGF1/PIP3-AKT pathway), and SB431542 (an inhibitor of the TGF-β/Activin A-SMAD pathway). *NANOG* tdTomato PC-iPS cells were treated continuously with inhibitors for three passages. Light microscopy showed that *NANOG* tdTomato PC-iPS cells were dome-shaped in all the groups (Fig. 5A, a). Fluorescence microscopy showed that only SB431542 detectably inhibited *NANOG* tdTomato expression (Fig. 5A, b). Flow cytometry analysis showed that the percentage of SB431542-treated cells expressing tdTomato was 4.53%, while over 95% of the cells receiving other inhibitors (or no treatment) expressed the tdTomato reporter (Fig. 5A, c). RT-PCR analysis also showed that expression of *NANOG* was the lowest in SB431542-treated cells, while *NANOG* expression in the other treatment groups was unchanged (Fig. 5A, d).

BMP4 supports the self-renewal of mouse pluripotent stem cells [25], but it is not known if it regulates the pluripotency of pig PSCs. To test the effects of BMP4, *NANOG* tdTomato PC-iPS cells were cultured with feeder cells for three continuous passages in the presence of hBMP4 or its antagonist Noggin. By the third passage, light microscopy showed that most of the cells treated with 10 ng/mL hBMP had differentiated, but cells in the control and antagonist groups had not (Fig. 5B, a). Fluorescence microscopy also showed that cells treated with 10 ng/mL hBMP had markedly lower expression of *NANOG* tdTomato, compared with cells cultured in T1 alone and cells receiving antagonists (Fig. 5B, b). Flow cytometry showed that 65.9% of cells treated with 10 ng/mL hBMP were positive for tdTomato. In contrast, the T1 and antagonist groups were over 85% tdTomato positive (Fig. 5B, c). Finally, treatment with hBMP4 (10 ng/ml) resulted in expression of *NANOG* mRNA being downregulated relative to the T1 control, but with hBMP4 (5 ng/ml) and Noggin, expression of *NANOG* mRNA was unchanged (Fig. 5B, d). In conclusion, inhibitors targeting the LIF/IL-6 STAT-JNK3, bFGF/ERK, IGF1/PIP3-AKT, TGF-β/Activin A, and BMP4-SMAD pathways did not affect *NANOG* expression, whereas the TGF-β/Activin A pathway inhibitor SB431542 completely abolished *NANOG* expression.

### Activation of NANOG expression by Activin A after SB431542 treatment

**Fig. 6:**
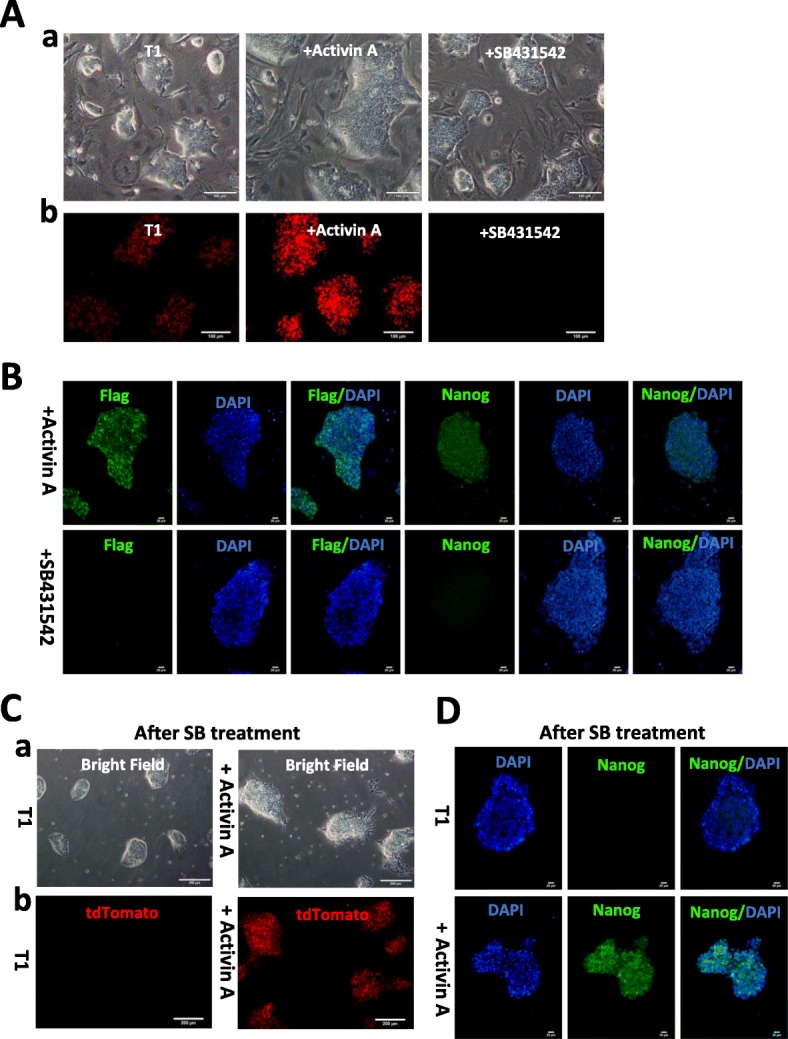
Rescue of NANOG expression after SB431542 treatment. **A** Optical and fluorescence microscopy showing NANOG expression in *NANOG* tdTomato knock-in positive cells grown in T1 alone, T1 + Activin A, and T1 + SB4316542. (a) Light microscopy showed that all cells were dome-shaped; (b) examination by fluorescence microscopy showed that cells treated with Activin A expressed more fluorescence than the controls, while those treated with SB431542 expressed no fluorescence. Scale bar 100 μm. **B** Immunocytological analysis of *NANOG* tdTomato knock-in positive cells grown in T1 + Activin A and T1 + SB4316542. FLAG and NANOG proteins were expressed in the Activin A-treated cells, while they were not detectable in T1 + SB4316542. Scale bars 20 μm. **C** Rescue of NANOG tdTomato expression after SB431542 treatment. (a) Light microscopy showed that NANOG tdTomato-positive PC-iPS cells cultured in T1 or T1 + Activin A were dome-shaped. (b) NANOG tdTomato expression was rescued only in cells cultured in T1 + Activin A. Scale bar 200 μm. **D** Immunocytological analysis shows that NANOG expression was seen only in cells cultured in T1 + Activin A after SB4316542 treatment. Scale bar 20 μm

To further test the effects of Activin A and its inhibitor SB431542 on NANOG expression, cells grown in T1 medium were treated with Activin A, SB431542, or remained untreated. Light microscopy showed that all cells were dome-shaped (Fig. 6A, a). Examination by fluorescence microscopy showed that cells treated with Activin A showed more fluorescent NANOG than the controls, while those treated with SB431542 showed no fluorescent NANOG (Fig. 6A, b). Immunocytochemical tests confirmed that the FLAG and NANOG proteins were expressed in the Activin A-treated cells but were undetectable in the SB431542-treated cells (Fig. 6B). To test whether Activin A can rescue NANOG expression after SB431542 treatment, SB431542-treated cells were transferred to a feeder-free culture system and divided two groups, one receiving T1 alone, and the other T1 and Activin A. After incubation for 3 days, light microscopy showed that NANOG tdTomato PC-iPS in T1 and T1 + Activin A were dome-shaped (Fig. 6C, a). Florescence microscopy showed that only in cells treated with T1 + Activin A was NANOG tdTomato expression rescued (Fig. 6C, b). Immunocytochemical tests also confirmed that the cells treated with Activin A expressed NANOG, while expression in T1 control cells was also absent (Fig. 6D). We conclude that Activin A rescues NANOG expression after SB431542 treatment in the feeder-free culture system.

### Differential genes expression in cells treated with Activin A and SB431542

**Fig. 7:**
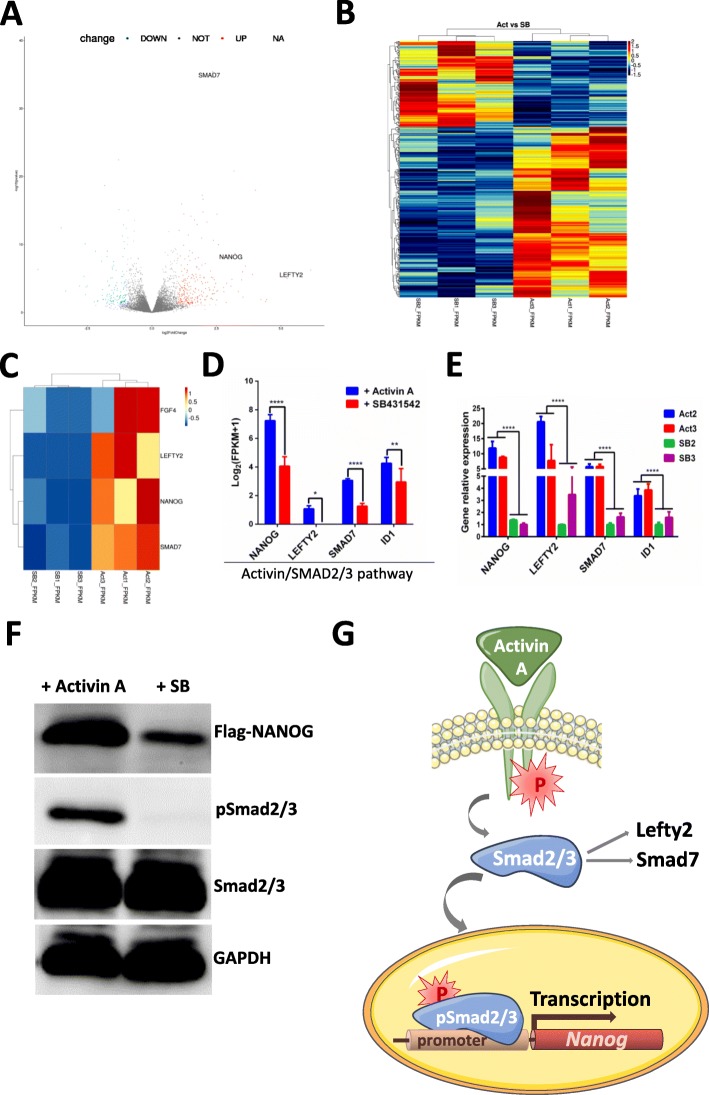
RNA-Seq analysis of cells treated with Activin A and SB431542. **a** Volcano plot showing expression values for genes in cells treated with Activin A versus SB431542. Genes represented by gray dots did not meet the criteria for differential expression. (log_2_[fold-change ≥1]; adjusted *p* value ≤ 0.05). (*n* = 3). **b** Heat map of the clustering analysis for differentially expressed genes in cells treated with Activin A versus SB431542. Adjst *p*<0.05 (*n* = 3). **c** Heat map showing expression of Activin/SMAD target genes. Adjst *p*<0.05 (*n* = 3). **d** Expression [log_2_(FPKM+1)] of Activin A/SMAD pathway target genes. **e** RT-PCR analysis of SMAD2/3 target gene expression. **f** Western blot analysis of selected proteins from cells grown with Activin A and SB431542. GAPDH was used as a loading control. **g** Model of the mechanism for Activin A regulation of *NANOG*. ****< 0.0001; ***< 0.001; **< 0.01, *< 0.5; ns, not significant

To better understand the effects of Activin A and SB431542, we examined global mRNA expression using high-throughput RNA sequencing to identify genes that were differentially expressed in response to these reagents. Genes were considered to be differentially expressed if |log_2_[fold-change]| ≥ 1 (Activin A-treated / SB431542-treated) with an adjusted *p* value<0.05. Using these criteria, 245 genes were classified as more abundant and 123 genes were less abundant in cells treated with Activin A versus cells treated with SB431542 (Additional file 9: Table S5; Fig. 7a)**.** Clustering analysis indicated that a large number of genes respond differently to the two treatments (Fig. 7b). KEGG pathway analysis (using adjusted *p* value<0.05) (Additional file 10: Table S6) strongly suggested involvement of the TGF-β signaling pathway (Additional file 11: Figure S5). Clustering analysis shows that Activin/SMAD pathway-related genes were upregulated in cells treated with Activin A vs. cells treated with SB431542 (Fig. 7c). Figure 7d shows the increases in expression of Smad2/3 target genes *NANOG*, *LEFTY2*, *SMAD7*, and *ID1.* In order to confirm that Activin A activates *NANOG* and that SB431542 inhibits *NANOG* through the Smad2/3 pathway, we measured the expression of Smad2/3 target genes using RT-PCR. The experiments confirmed that the levels of *NANOG*, *LEFTY2*, *SMAD7*, and *ID1* mRNA were higher in cells treated with Activin A than in cells treated with SB431542 (Fig. 7e). Western blots confirmed that NANOG protein is more abundant in Activin A-treated cells than in those treated with SB431542. Cells in both treatment groups expressed Smad2/3 protein, but pSmad2/3 protein was absent in cells treated with SB4315423 (Fig. 7f). In conclusion, Activin A directly regulates the expression of *NANOG* via pSmad2/3 in pig iPS cells.

## Discussion

*NANOG* knock-in reporter PSCs have been used to study *NANOG* regulation in mice and humans. In this study, pig *NANOG* tdTomato knock-in positive reporter iPS cells were used to test the effects of several cytokines, their corresponding inhibitors, and the components of a primary culture system (LCDMV) on *NANOG* expression.

In the analysis of LCDMV medium, removal of DIM caused cells to differentiate and assume a flatted morphology. Similar results have been obtained using EPS cells from mice and humans [44]. Therefore, regulation of pluripotency by DIM appears to be conserved in mice, humans, and pigs. When CHIR was removed, *NANOG* expression decreased. CHIR, a component in 2i culture systems, plays an important role in maintaining mouse ES self-renewal [45]. Vc promotes iPS generation in mice and humans [46]. In our study, Vc was also vital for maintaining NANOG tdTomato expression. CDMV can sustain *NANOG* expression in feeder cells for long periods. However, when cells were transferred to a feeder-free system, NANOG tdTomato expression decreased, and by the third passage, tdTomato protein was not detectable by flow cytometry or fluorescence microscopy. Feeder cells are therefore necessary for NANOG tdTomato expression in pig PC-iPS cells without additional cytokines.

Heterogeneous NANOG is expressed in mouse embryonic stem cells [7]. But, patterns of NANOG in pig pluripotent stem cells (PSCs) from pre-implantation embryos are different from pig iPS cells. NANOG is highly expressed in pig ES cells from ICM/epiblast [13] and in pig EPS cells [12]. The NANOG expression of pig iPS cells in doxycycline-inducible system with 4 factors (OSKC) was low [36]. Pig iPS cells with doxycycline-inducible piggyBac (PB) expression vectors encoding porcine 4 factors (OSKC) NANOG expression were absent [34]. Our previous work showed that pig PC-iPS with 4 factors (OSKC), NANOG is heterogeneously expressed in LCDMV culture medium [14]. Others report that in pig iPS cells generated by episomal vectors [32] and lentivirus vectors [47] separately containing 6 factors (OKSM+NANOG+LIN28) NANOG is expressed, but exogenous *NANOG* factor has not been silenced. Activation of *NANOG* is necessary for establishing authentic pig iPS cells. Our NANOG tdTomato knock-in reporter positive piPS cells exhibited higher expression of the pluripotent genes *NANOG*, *TFCP2L1*, *CDH1*, *KLF2*, and *KLF5*, as well as higher NANOG protein expression than WT PC-iPS cells. Whether NANOG tdTomato knock-in reporter positive PC-iPS cells can meet the gold standard quality of pig iPS cells needs to be investigated using in vivo chimera tests.

LIF and BMP4 maintain pluripotency of mESCs [25], but hESCs do not depend on the LIF/STAT pathway [48]. Treatment with BMP4 promotes hESC differentiation [26]. We found that pig NANOG tdTomato PC-iPS cells were similar to hES cells in their response to BMP4 and do not depend on the LIF signal pathway. While the bFGF/ERK pathway maintains hESC [49] and mouse EpiSC [22, 50] pluripotency, it promotes *NANOG* tdTomato knock-in positive PC-iPS cell differentiation. This suggests that the bFGF/ERK pathway does not directly target *NANOG* in pig iPS cells. Activation of the AKT pathway is sufficient for maintaining the pluripotency of mouse cells [51]. IGF-1 and IGF-2 activate the PI3K/AKT pathway, thus maintaining human ESCs [28, 52]. This result contrasts with our results using pig NANOG tdTomato PC-iPS cells. However, when PI3K/AKT signaling is inhibited by LY294002, pig NANOG tdTomato PC-iPS cells proliferation decreases, as is also the case for human ES cells [49]. Thus, the PI3K/AKT pathway appears to be conserved in mice, and humans, but its function differs in pigs. Based on our cytokine screen in the absence of feeder cells, only Activin A activated NANOG tdTomato expression. This is also observed in humans and mice. *NANOG* is a direct target of the Activin A-SMAD2/3 pathway that maintains human [24, 53] and mouse ESC [54] pluripotency. Activin A can also maintain human ESC pluripotency in the absence of feeder layers [55], which is consistent with our results using pig cells. In conclusion, the Activin-SMAD pathway appears to directly regulate *NANOG* and is conserved among mice, pigs, and humans.

## Conclusions

In this study, pig *NANOG* tdTomato knock-in reporter iPS cells were employed to screen the key pathways involved in *NANOG* regulation. The Activin A/SMAD pathway directly regulates *NANOG* and appears to be conserved among mice, pigs, and humans. In contrast, the LIF/JNK-STAT, IGF/PIP3-AKT, FGF/ERK, and BMP4/SMAD pathways did not affect pig *NANOG* regulation in pig iPS cells, which is contrary to results obtained in mice and humans (Additional file 12). Therefore, Activin A may be used for de novo isolation of pig ESCs from pre-implantation embryos.

## Additional files

Additional file1 Table S1. Primers used for *NANOG* knock-in reporter construction and RT-PCR. (DOCX 19 kb)

Additional file 2 Figure S1. Vector structures. A. *NANOG* sgRNA vector.. B. *NANOG HMEJ* donor vector. (PDF 158 kb)

Additional file 3 Figure S2. Molecular validation of *NANOG* tdTomato knock-in reporter positive PC-iPS. A. Overview of process used to generate *NANOG* tdTomato knock-in positive PC-iPS cells. B. Genotyping by PCR analysis at the 5′ and 3′ junctions of *NANOG* tdTomato knock-in constructs. C. knock-in positive PC-iPS cells were positive to AP staining, Scale bar 50 μm. (PDF 262 kb)

Additional file 4 : Table S2. Detail of genes expression of clustering Nanog, pEPS and embryos.

Additional file 5 : Figure S3. Transcriptome of NANOG tdTomato Knock-in reporter positive PC-iPS versus WT PC-iPS. A, Volcano plot showing distribution of fold-change values (x-axis) and log_10_ (adjusted *p*-values) (y-axis). A gene was defined as differentially expressed if its fold-change value |(log_2_[fold-change])|, calculated as *NANOG* tdTomato/WT PC-iPS, was 1 or more, with an adjusted p-value ≤0.05 (*n* = 3). Genes meeting these criteria are shown as red dots (if more abundant), blue dots (if less abundant), and gray (if relatively unchanged). Genes that are discussed in detail in the text have been labeled. B, Heat map of the clustering analysis of gene expression in *NANOG* tdTomato knock-in positive and WT PC-iPS cells. The color scale represents the fold-change in expression as |(log_2_[fold-change])|.

Additional file 6 : Table S3. Differentially expressed genes (DEGs) identified using RNA-Seq data obtained from *NANOG* tdTomato knock-in positive PC-iPS cells vs. WT PC-iPS cells. (log_2_[fold-change ≥1]; adjusted p-value ≤0.05).

Additional file 7 : Table S4. KEGG pathway enrichment analysis for differentially expressed genes identified using RNA-Seq data obtained from *NANOG* tdTomato knock-in positive PC-iPS cells vs. WT PC-iPS cells.

Additional file 8 : Figure S4. KEGG pathway analyses of differentially expressed genes identified by RNA-Seq in *NANOG* tdTomato knock-in positive PC-iPS cells vs. PC-iPS cells.

Additional file 9 : Table S5. Differentially expressed genes (DEGs) identified using RNA-Seq data obtained from *NANOG* tdTomato knock-in positive PC-iPS cells treated with Activin A or SB431542. (log_2_[fold-change ≥1]; adjusted p-value ≤0.05).

Additional file 10 : Table S6. KEGG pathway enrichment analysis for differentially expressed genes (DEGs) identified using RNA-Seq data obtained from *NANOG* tdTomato knock-in positive PC-iPS cells treated with Activin A or SB431542.

Additional file 11 : Figure S5. KEGG pathway enrichment analysis of differentially expressed genes identified by RNA-Seq in *NANOG* tdTomato knock-in positive PC-iPS cells in the presence of Activin A or SB431542.

Additional file 12 : Model of cytokine regulation of *NANOG* in mice, humans, and pigs.

## Abbreviations

Act: Activin A
AP: Alkaline phosphatase
BMP4: Bone morphogenetic proteins
CHIR: CHIR99021
Chr: Chromosome
DIM: (S)-(+)-dimethindene maleate
EPSCs: Expanded pluripotent stem cells
ESCs: Embryonic stem cells
FGF: Fibroblast growth factor
ICM: Inner cell mass
IGF1: Insulin-like growth factor 1
IL6: Interleukin 6
iPSCs: Induced pluripotent stem cells
LCDMV: Medium with LIF, CHIR, DIM, MIH, and Vc
LIF: Leukemia inhibitory factor
MIH: Minocycline hydrochloride
NHEJ: Non-homologous end joining
OSKC: OCT4, SOX2, KLF4, c-Myc
PAM: Protospacer adjacent motif
PIP3: Phosphoinositide 3-kinase
PSCs: Pluripotent stem cells
SB: SB431542
sgRNA: Single-guide RNA
T1: Medium with CHIR, DIM, MIH, and Vc
Vc: Vitamin C
WT: Wild-type

## References

[1] Evans MJ, Kaufman MH (1981). Establishment in culture of pluripotential cells from mouse embryos. Nature.

[2] Thomson JA, Itskovitz-Eldor J, Shapiro SS, Waknitz MA, Swiergiel JJ, Marshall VS, Jones JM (1998). Embryonic stem cell lines derived from human blastocysts. Science.

[3] Boyer LA, Mathur D, Jaenisch R (2006). Molecular control of pluripotency. Curr Opin Genet Dev.

[4] Boyer LA, Lee TI, Cole MF, Johnstone SE, Levine SS, Zucker JP, Guenther MG, Kumar RM, Murray HL, Jenner RG (2005). Core transcriptional regulatory circuitry in human embryonic stem cells. Cell.

[5] Torres J, Watt FM (2008). Nanog maintains pluripotency of mouse embryonic stem cells by inhibiting NFkappaB and cooperating with Stat3. Nat Cell Biol.

[6] Pan G, Thomson JA (2007). Nanog and transcriptional networks in embryonic stem cell pluripotency. Cell Res.

[7] Chambers I, Silva J, Colby D, Nichols J, Nijmeijer B, Robertson M, Vrana J, Jones K, Grotewold L, Smith A (2007). Nanog safeguards pluripotency and mediates germline development. Nature.

[8] Hyslop L, Stojkovic M, Armstrong L, Walter T, Stojkovic P, Przyborski S, Herbert M, Murdoch A, Strachan T, Lako M (2005). Downregulation of NANOG induces differentiation of human embryonic stem cells to extraembryonic lineages. Stem Cells.

[9] Singh AM, Hamazaki T, Hankowski KE, Terada N (2007). A heterogeneous expression pattern for Nanog in embryonic stem cells. Stem Cells.

[10] Ramos-Ibeas P, Sang F, Zhu Q, Tang WWC, Withey S, Klisch D, Wood L, Loose M, Surani MA, Alberio R (2019). Pluripotency and X chromosome dynamics revealed in pig pre-gastrulating embryos by single cell analysis. Nat Commun.

[11] Zhang X, Xue B, Li Y, Wei R, Yu Z, Jin J, Zhang Y, Liu Z: A novel chemically defined serum- and feeder-free medium for undifferentiated growth of porcine pluripotent stem cells. J Cell Physiol. 2019. 10.1002/jcp.28185. [Epub ahead of print] 10.1002/jcp.28185 30701540

[12] Gao X, Nowak-Imialek M, Chen X, Chen D, Herrmann D, Ruan D, Chen ACH, Eckersley-Maslin MA, Ahmad S, Lee YL (2019). Establishment of porcine and human expanded potential stem cells. Nat Cell Biol.

[13] Choi KH, Lee DK, Kim SW, Woo SH, Kim DY, Lee CK: Chemically Defined Media Can Maintain Pig Pluripotency Network In Vitro. Stem Cell Reports. 2019;13(1):221–34. 10.1016/j.stemcr.2019.05.028 PMC6626979 31257130

[14] Xu J, Yu L, Guo J, Xiang J, Zheng Z, Gao D, Shi B, Hao H, Jiao D, Zhong L (2019). Generation of pig induced pluripotent stem cells using an extended pluripotent stem cell culture system. Stem Cell Res Ther.

[15] Kumar RM, Cahan P, Shalek AK, Satija R, DaleyKeyser A, Li H, Zhang J, Pardee K, Gennert D, Trombetta JJ (2014). Deconstructing transcriptional heterogeneity in pluripotent stem cells. Nature.

[16] Smith RCG, Stumpf PS, Ridden SJ, Sim A, Filippi S, Harrington HA, MacArthur BD (2017). Nanog fluctuations in embryonic stem cells highlight the problem of measurement in cell biology. Biophys J.

[17] Miyanari Y, Torres-Padilla M-E (2012). Control of ground-state pluripotency by allelic regulation of Nanog. Nature.

[18] Xenopoulos P, Kang M, Puliafito A, Di Talia S, Hadjantonakis A-K (2015). Heterogeneities in Nanog expression drive stable commitment to pluripotency in the mouse blastocyst. Cell Rep.

[19] Filipczyk A, Marr C, Hastreiter S, Feigelman J, Schwarzfischer M, Hoppe PS, Loeffler D, Kokkaliaris KD, Endele M, Schauberger B (2015). Network plasticity of pluripotency transcription factors in embryonic stem cells. Nat Cell Biol.

[20] Faddah DA, Wang H, Cheng AW, Katz Y, Buganim Y, Jaenisch R (2013). Single-cell analysis reveals that expression of nanog is biallelic and equally variable as that of other pluripotency factors in mouse ESCs. Cell Stem Cell.

[21] Nichols J, Smith A (2009). Naive and primed pluripotent states. Cell Stem Cell.

[22] De Los AA, Loh YH, Tesar PJ, Daley GQ (2012). Accessing naive human pluripotency. Curr Opin Genet Dev.

[23] Brady JJ, Li M, Suthram S, Jiang H, Wong WH, Blau HM (2013). Early role for IL-6 signalling during generation of induced pluripotent stem cells revealed by heterokaryon RNA-Seq. Nat Cell Biol.

[24] Xu RH, Sampsell-Barron TL, Gu F, Root S, Peck RM, Pan G, Yu J, Antosiewicz-Bourget J, Tian S, Stewart R (2008). NANOG is a direct target of TGFbeta/activin-mediated SMAD signaling in human ESCs. Cell Stem Cell.

[25] Ying QL, Nichols J, Chambers I, Smith A (2003). BMP induction of id proteins suppresses differentiation and sustains embryonic stem cell self-renewal in collaboration with STAT3. Cell.

[26] Xu RH, Chen X, Li DS, Li R, Addicks GC, Glennon C, Zwaka TP, Thomson JA (2002). BMP4 initiates human embryonic stem cell differentiation to trophoblast. Nat Biotechnol.

[27] Amita M, Adachi K, Alexenko AP, Sinha S, Schust DJ, Schulz LC, Roberts RM, Ezashi T (2013). Complete and unidirectional conversion of human embryonic stem cells to trophoblast by BMP4. Proc Natl Acad Sci U S A.

[28] Bendall SC, Stewart MH, Menendez P, George D, Vijayaragavan K, Werbowetski-Ogilvie T, Ramos-Mejia V, Rouleau A, Yang J, Bosse M (2007). IGF and FGF cooperatively establish the regulatory stem cell niche of pluripotent human cells in vitro. Nature.

[29] Wang L, Schulz TC, Sherrer ES, Dauphin DS, Shin S, Nelson AM, Ware CB, Zhan M, Song CZ, Chen X (2007). Self-renewal of human embryonic stem cells requires insulin-like growth factor-1 receptor and ERBB2 receptor signaling. Blood.

[30] Chen L, Khillan JS (2010). A novel signaling by vitamin A/retinol promotes self renewal of mouse embryonic stem cells by activating PI3K/Akt signaling pathway via insulin-like growth factor-1 receptor. Stem Cells.

[31] Nguyen TT, Sheppard AM, Kaye PL, Noakes PG (2007). IGF-I and insulin activate mitogen-activated protein kinase via the type 1 IGF receptor in mouse embryonic stem cells. Reproduction.

[32] Du X, Feng T, Yu D, Wu Y, Zou H, Ma S, Feng C, Huang Y, Ouyang H, Hu X (2015). Barriers for deriving transgene-free pig iPS cells with episomal vectors. Stem Cells.

[33] Zhang Y, Wei C, Zhang P, Li X, Liu T, Pu Y, Li Y, Cao Z, Cao H, Liu Y (2014). Efficient reprogramming of naive-like induced pluripotent stem cells from porcine adipose-derived stem cells with a feeder-independent and serum-free system. PLoS One.

[34] Liu K, Ji G, Mao J, Liu M, Wang L, Chen C, Liu L (2012). Generation of porcine-induced pluripotent stem cells by using OCT4 and KLF4 porcine factors. Cell Reprogram.

[35] Vassiliev I, Vassilieva S, Beebe LF, Harrison SJ, McIlfatrick SM, Nottle MB (2010). In vitro and in vivo characterization of putative porcine embryonic stem cells. Cell Reprogram.

[36] Ma Y, Yu T, Cai Y, Wang H (2018). Preserving self-renewal of porcine pluripotent stem cells in serum-free 3i culture condition and independent of LIF and b-FGF cytokines. Cell Death Discov.

[37] Montserrat N, Bahima EG, Batlle L, Hafner S, Rodrigues AM, Gonzalez F, Izpisua Belmonte JC (2011). Generation of pig iPS cells: a model for cell therapy. J Cardiovasc Transl Res.

[38] Kim D, Langmead B, Salzberg SL (2015). HISAT: a fast spliced aligner with low memory requirements. Nat Methods.

[39] Liao Y, Smyth GK, Shi W (2014). featureCounts: an efficient general purpose program for assigning sequence reads to genomic features. Bioinformatics.

[40] Pertea M, Kim D, Pertea GM, Leek JT, Salzberg SL (2016). Transcript-level expression analysis of RNA-seq experiments with HISAT, StringTie and Ballgown. Nat Protoc.

[41] Love MI, Huber W, Anders S (2014). Moderated estimation of fold change and dispersion for RNA-seq data with DESeq2. Genome Biol.

[42] Yu G, Wang LG, Han Y, He QY (2012). clusterProfiler: an R package for comparing biological themes among gene clusters. OMICS.

[43] Kong Q, Yang X, Zhang H, Liu S, Zhao J, Zhang J, Weng X, Jin J, Liu Z. Lineage specification and pluripotency revealed by transcriptome analysis from oocyte to blastocyst in pig. The FASEB Journal. 2019;34(1):691–705. 10.1096/fj.201901818RR 31914626

[44] Yang Y, Liu B, Xu J, Wang J, Wu J, Shi C, Xu Y, Dong J, Wang C, Lai W (2017). Derivation of pluripotent stem cells with in vivo embryonic and extraembryonic potency. Cell.

[45] Ying QL, Wray J, Nichols J, Batlle-Morera L, Doble B, Woodgett J, Cohen P, Smith A (2008). The ground state of embryonic stem cell self-renewal. Nature.

[46] Esteban MA, Wang T, Qin B, Yang J, Qin D, Cai J, Li W, Weng Z, Chen J, Ni S (2010). Vitamin C enhances the generation of mouse and human induced pluripotent stem cells. Cell Stem Cell.

[47] Fukuda T, Tani T, Haraguchi S, Donai K, Nakajima N, Uenishi H, Eitsuka T, Miyagawa M, Song S, Onuma M (2017). Expression of six proteins causes reprogramming of porcine fibroblasts into induced pluripotent stem cells with both active X chromosomes. J Cell Biochem.

[48] Daheron L, Opitz SL, Zaehres H, Lensch MW, Andrews PW, Itskovitz-Eldor J, Daley GQ (2004). LIF/STAT3 signaling fails to maintain self-renewal of human embryonic stem cells. Stem Cells.

[49] Li J, Wang G, Wang C, Zhao Y, Zhang H, Tan Z, Song Z, Ding M, Deng H (2007). MEK/ERK signaling contributes to the maintenance of human embryonic stem cell self-renewal. Differentiation.

[50] Hanna JH, Saha K, Jaenisch R (2010). Pluripotency and cellular reprogramming: facts, hypotheses, unresolved issues. Cell.

[51] Watanabe S, Umehara H, Murayama K, Okabe M, Kimura T, Nakano T (2006). Activation of Akt signaling is sufficient to maintain pluripotency in mouse and primate embryonic stem cells. Oncogene.

[52] Singh AM, Reynolds D, Cliff T, Ohtsuka S, Mattheyses AL, Sun Y, Menendez L, Kulik M, Dalton S (2012). Signaling network crosstalk in human pluripotent cells: a Smad2/3-regulated switch that controls the balance between self-renewal and differentiation. Cell Stem Cell.

[53] Tomizawa M, Shinozaki F, Sugiyama T, Yamamoto S, Sueishi M, Yoshida T (2011). Activin A maintains pluripotency markers and proliferative potential of human induced pluripotent stem cells. Exp Ther Med.

[54] Vallier L, Mendjan S, Brown S, Chng Z, Teo A, Smithers LE, Trotter MW, Cho CH, Martinez A, Rugg-Gunn P (2009). Activin/nodal signalling maintains pluripotency by controlling Nanog expression. Development.

[55] Beattie GM, Lopez AD, Bucay N, Hinton A, Firpo MT, King CC, Hayek A (2005). Activin A maintains pluripotency of human embryonic stem cells in the absence of feeder layers. Stem Cells.

