# Supplementary material for: A cytokine screen using CRISPR-Cas9 knock-in reporter pig iPS cells reveals that Activin A regulates NANOG
Source: Stem Cell Res Ther. 2020 Feb 18;11:67. doi: 10.1186/s13287-020-1588-z (PMC7029561; doi:10.1186/s13287-020-1588-z)
Supplement: Supplementary file 1 — Table S1. Primers used for NANOG knock-in reporter construction and RT-PCR. (DOCX 19 kb) [file 13287_2020_1588_MOESM1_ESM.docx]

**Table1. Primer List for CRISP/CAS9 knock in reporter construction and RT-PCR**

| **Primer for Donor construction** | |
| --- | --- |
| **Primer** | **Sequence** |
| NANOG Raw F | CTTACTGACTCCACCGGCAC |
| NANOG Raw R | ATGAACCAGTCACACTGGCAGG |
| NANOG 5' Arm 1 F | ATCTTCAGGCTGTATGTTCATGGTGCAGATGTCAGCTGCTGAGAC |
| NANOG 5' Arm 1 Mut R | CATATCTTCAGGCTGTATGTTCATAGAGTAATTTGGGAAT |
| NANOG 3' Arm 1 F | CGATCATTTTATTTTTTTAAAAAATTTTATTGGAGTATAGTTGATTTACA |
| NANOG 3' Arm 1 R | ATCTTCAGGCTGTATGTTCATGGAGCACAGAAGGCATGAGACCAG |
| NANOG 5' Arm 2 F | GAAAAATAAACAAATAGGGGTTCAAGCTTATCTTCAGGCTGTATGTTCATGGTG |
| NANOG 5' Arm 2 R | CGTCGTCATCCTTGTAATCCATATCTTCAGGCTGTATGTTCATAGAGTAATTTGG |
| NANOG 5' Arm 3 F | GAAAAATAAACAAATAGGGGTTCAAGCTTATCTTCAGGCTGTATGTTCATGGTG |
| NANOG 5'Arm 3 R | TTATCGTCGTCATCCTTGTAATCGCCCTTATCGTCGTCATCCTTGTAATCCATATCTTC |
| NANOG 3' Arm 2 F | AGTTATATCGATCGATCATTTTATTTTTTTAAAAAATTTTATTGGAGTATAGTTGATT |
| NANOG 3' Arm 2 R | AGCTATGACCATGGCTCTAGAATCTTCAGGCTGTATGTTCATGGAG |
| TdTomato-Puro 1F | GTTTCAGGAAGCGGAGCTACTAACTTCAGCCTGCTGAAGCA |
| TdTomato-Puro 1R | GATCGATCGATATAACTTCGTATAGCATACATTAT |
| TdTomato-Puro 2F | ATAAGGGCGATTACAAGGATGACGACGATAAGGGAGATTACAAGGATGACGACGATAAGGTTTCAGGAAGCGGAGCTACTAACTTCAG |
| TdTomato-Puro 2R | ATGATCGATCGATATAACTTCGTATAGCAT |
| **Primer for sgRNA construction** | |
| **Primer** | **Sequence** |
| NANOG sgRNA F | ACCGATCTTCAGGCTGTATGTTCA |
| NANOG sgRNA R | AAACTGAACATACAGCCTGAAGAT |
| **Primer for Genotyping** | |
| **Primer** | **Sequence** |
| Chr1 5' Test F | CCTGAGGTTTATGGGCCTGA |
| Chr1 5' Test R | TGAGGTCCCACTGTAGAGCA |
| Chr1 3' Test F | GCTAGCTTACCATGACCGAGTACAA |
| Chr1 3' Test R | TGCTTCTAACTAGGAAGGAGAATC |
| Chr5 5' Test F | TGTCCATTGCTGAAGCATGTAAT |
| Chr5 5' Test R | GAAGTTAGTAGCTCCGCTTCCTG |
| Chr5 3' Test F | GCTAGCTTACCATGACCGAGTACAA |
| Chr5 3' Test R | CTCTACCACCCATTATTCGCCT |

| **Primers for RT-PCR** | |
| --- | --- |
| **Primer** | **Sequence** |
| Endo-Nanog-F | CCTACAATCCAGCTCTTTGG |
| Endo-Nanog-R | CTCAGGCATTGGTGAAGATT |
| Endo-Oct4-F | CTTCACCACCCTGTACTCCTC |
| Endo-Oct4-R | GCTTCTCTCCCTAGCTCACC |
| Endo-Sox2-F | CATCAACGGTACACTGCCTCTC |
| Endo-Sox2-R | ACTCTCCTCCCATTTCCCTCTTT |
| CDH1-F  CDH1-R | GCAATCACCTCACGGGAAT  TTATCAGCACCCACGCAA |
| TCFP2L1-F | TGCACGAAGAGACCTTGACC |
| TCFP2L1-R | CGCGGATGGTACTCTTCACA |
| LEFTY2-F  LEFTY2-R  Smad7-F  Smad7-R  ID1-F  ID1-R | CCACGTGAGGGCCCAGTA  TCCATGTCGAACACCAGCAG  TACTGGGAGGAGAAGACGAGAGTG  TGGCTGACTTGATGAAGATGGG  CTCGACGAACAGCAGGTGAA  CAGATTGGAGGGGACAAGATT |
