# Supplementary figures and images for: A cytokine screen using CRISPR-Cas9 knock-in reporter pig iPS cells reveals that Activin A regulates NANOG
Source: Stem Cell Res Ther. 2020 Feb 18;11:67. doi: 10.1186/s13287-020-1588-z (PMC7029561; doi:10.1186/s13287-020-1588-z)

**A**

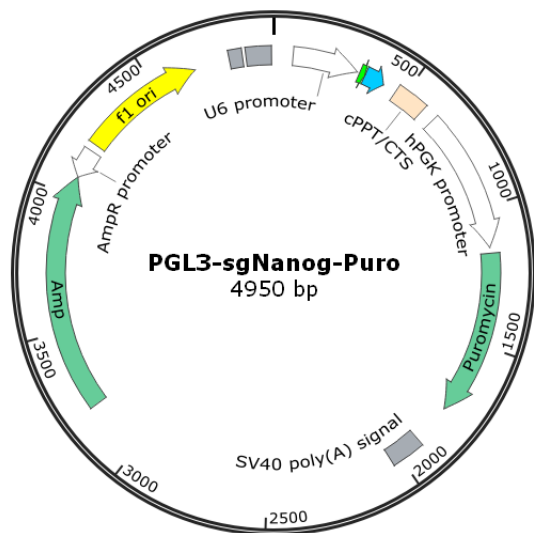

**B**

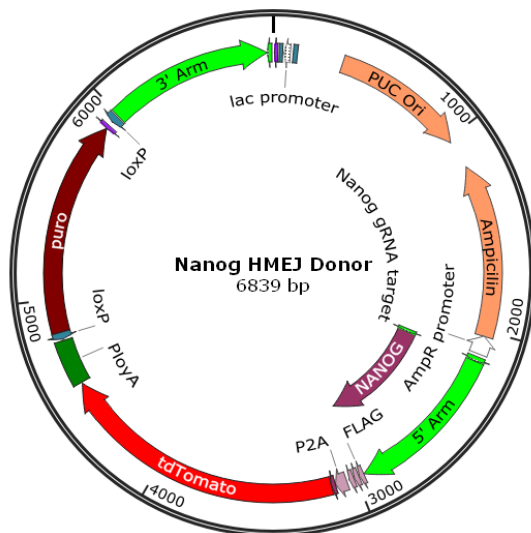

Supplement: Supplementary file 2 — Figure S1. Vector structures. A. NANOG sgRNA vector.. B. NANOG HMEJ donor vector. (PDF 158 kb) [file 13287_2020_1588_MOESM2_ESM.pdf]

**A**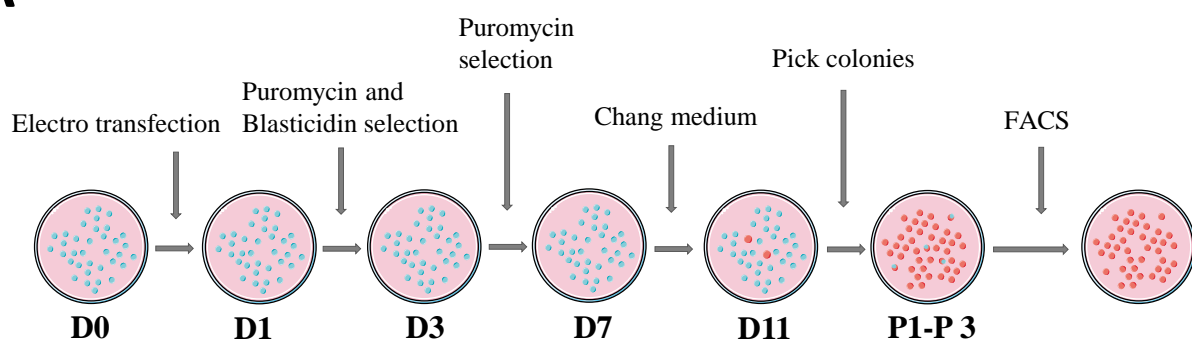**B**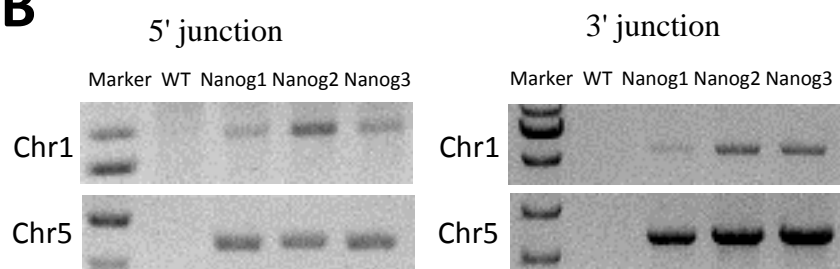**C**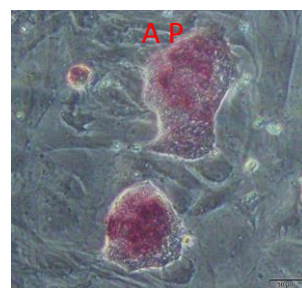

Supplement: Supplementary file 3 — Figure S2. Molecular validation of NANOG tdTomato knock-in reporter positive PC-iPS. A. Overview of process used to generate NANOG tdTomato knock-in positive PC-iPS cells. B. Genotyping by PCR analysis at the 5′ and 3′ junctions of NANOG tdTomato knock-in constructs. C. knock-in positive PC-iPS cells were positive to AP staining, Scale bar 50 μm. (PDF 262 kb) [file 13287_2020_1588_MOESM3_ESM.pdf]

A

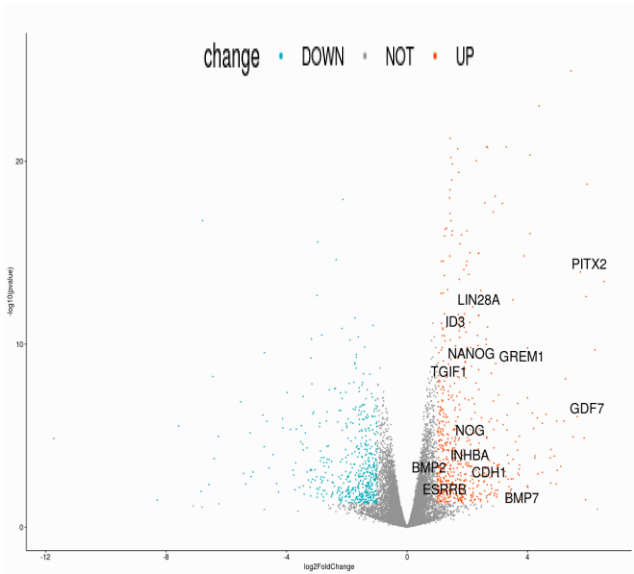

B

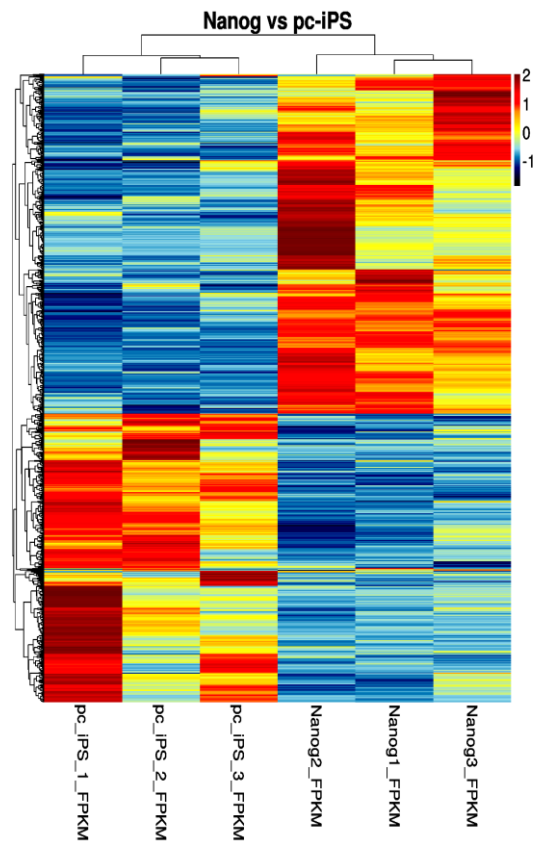

Supplement: Supplementary file 5 — : Figure S3. Transcriptome of NANOG tdTomato Knock-in reporter positive PC-iPS versus WT PC-iPS. A, Volcano plot showing distribution of fold-change values (x-axis) and log10 (adjusted p-values) (y-axis). A gene was defined as differentially expressed if its fold-change value |(log2[fold-change])|, calculated as NANOG tdTomato/WT PC-iPS, was 1 or more, with an adjusted p-value ≤0.05 (n = 3). Genes meeting these criteria are shown as red dots (if more abundant), blue dots (if less abundant), and gray (if relatively unchanged). Genes that are discussed in detail in the text have been labeled. B, Heat map of the clustering analysis of gene expression in NANOG tdTomato knock-in positive and WT PC-iPS cells. The color scale represents the fold-change in expression as |(log2[fold-change])|. [file 13287_2020_1588_MOESM5_ESM.pdf]

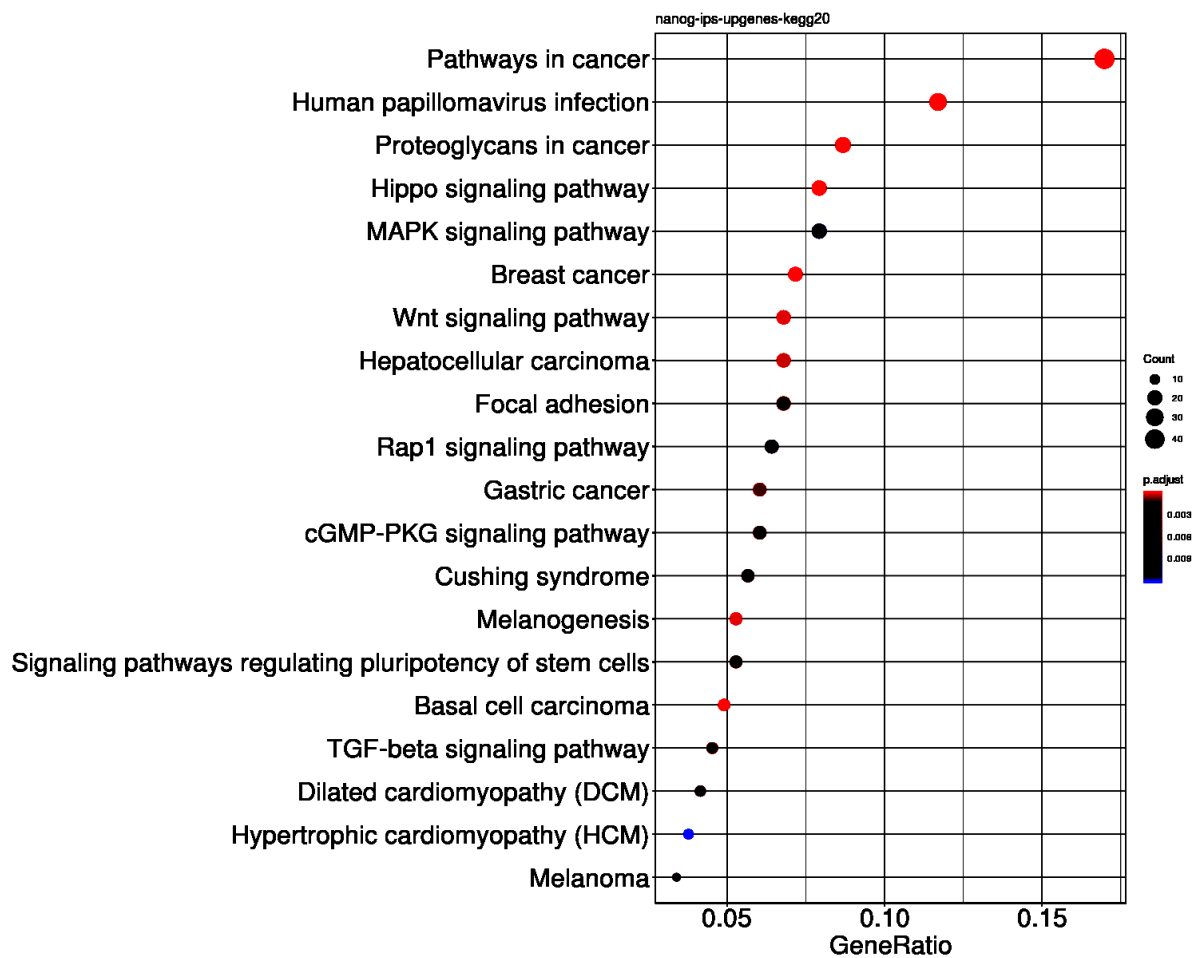

Supplement: Supplementary file 8 — : Figure S4. KEGG pathway analyses of differentially expressed genes identified by RNA-Seq in NANOG tdTomato knock-in positive PC-iPS cells vs. PC-iPS cells. [file 13287_2020_1588_MOESM8_ESM.pdf]

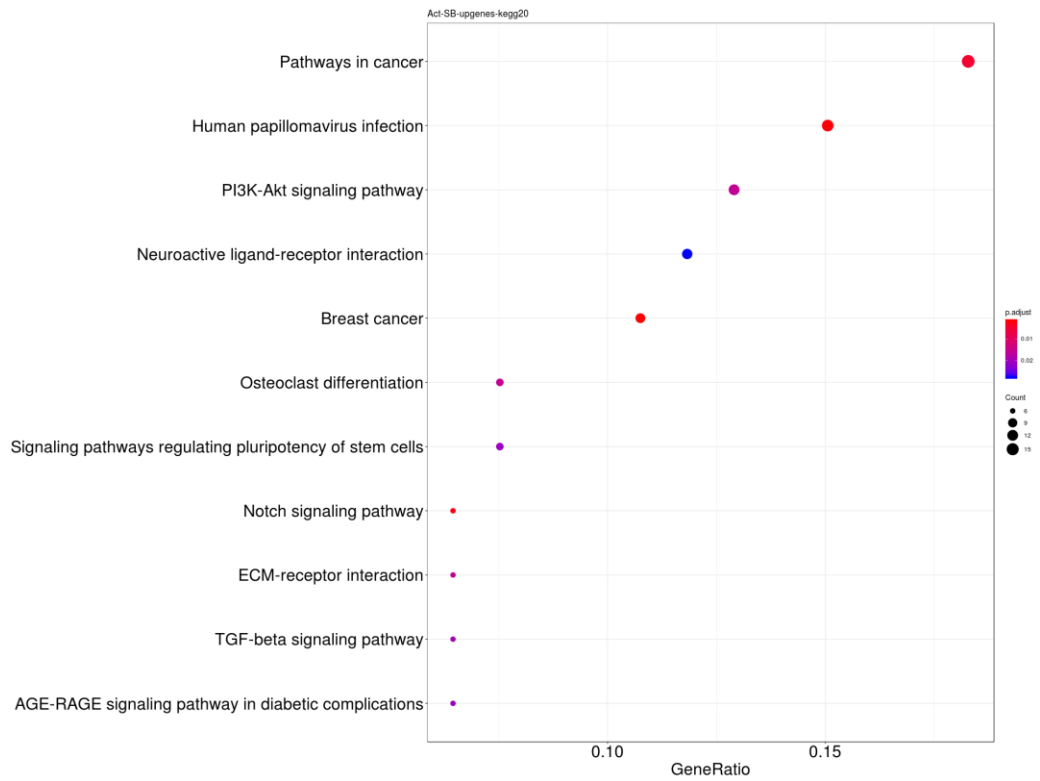

Supplement: Supplementary file 11 — : Figure S5. KEGG pathway enrichment analysis of differentially expressed genes identified by RNA-Seq in NANOG tdTomato knock-in positive PC-iPS cells in the presence of Activin A or SB431542. [file 13287_2020_1588_MOESM11_ESM.pdf]

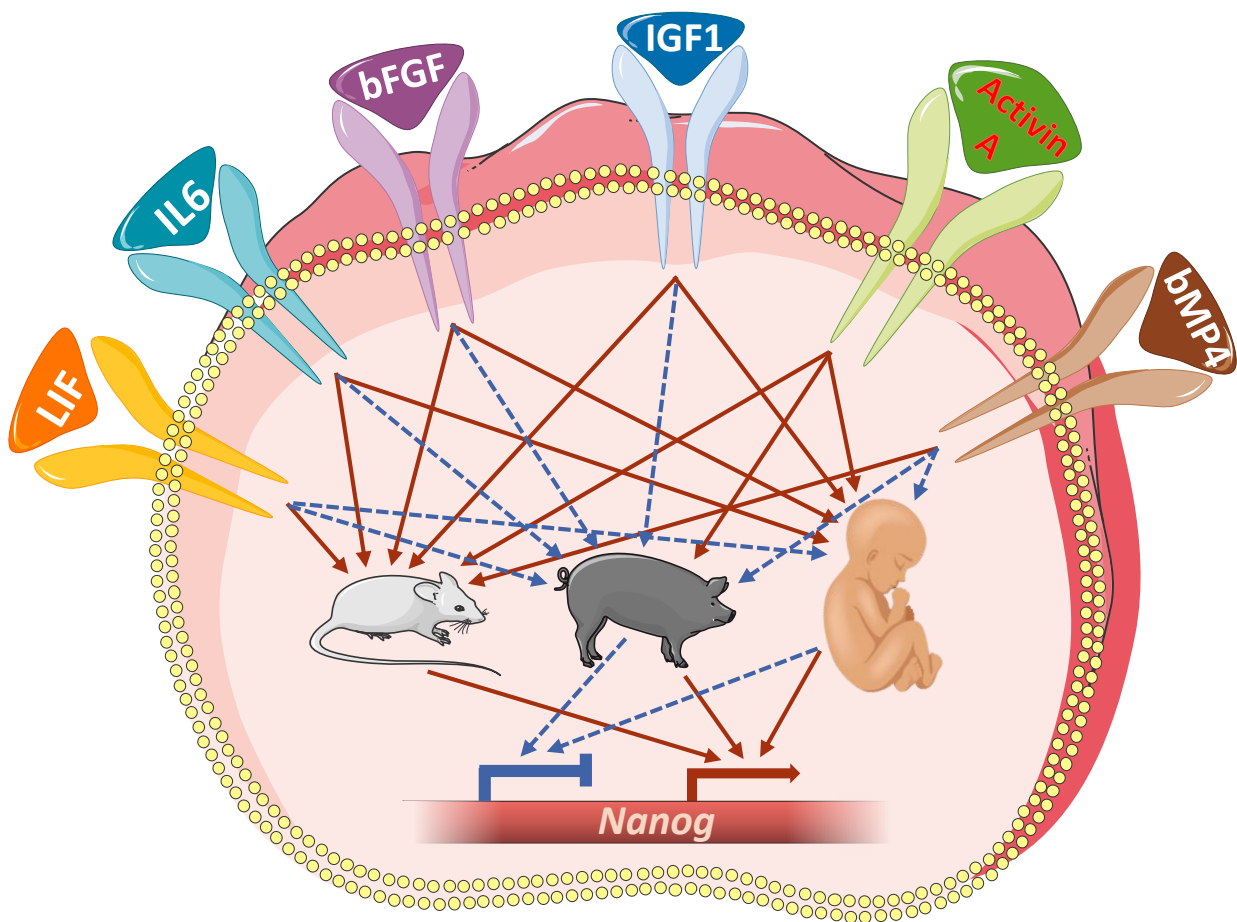

—————> **Activation of Nanog**  
 - - - - -> **No activation of Nanog**

Supplement: Supplementary file 12 — : Model of cytokine regulation of NANOG in mice, humans, and pigs. [file 13287_2020_1588_MOESM12_ESM.pdf]
